# Costs of shoulder pain and resource use in primary health care: a cost-of-illness study in Sweden

**DOI:** 10.1186/1471-2474-13-17

**Published:** 2012-02-10

**Authors:** Lena Virta, Pål Joranger, Jens Ivar Brox, Rikard Eriksson

**Affiliations:** 1 Medical Faculty, University of Oslo, Oslo, Norway; 2 Department of Faculty of Health, Nutrition and Management, Faculty of Health Sciences, Oslo and Akershus University College of Applied Sciences, Postbox 4, St. Olavsplass, 0130 Oslo, Norway; 3 Department of Orthopaedics, Oslo University Hospital, 0027 Oslo, Norway

## Abstract

**Background:**

Painful shoulders pose a substantial socioeconomic burden. A prospective cost-of-illness study was performed to assess the costs associated with healthcare use and loss of productivity in patients with shoulder pain in primary health care in Sweden.

**Methods:**

The study was performed in western Sweden, in a region with 24 000 inhabitants. Data were collected during six months from electronic patient records at three primary healthcare centres in two municipalities. All patients between 20 and 64 years of age who presented with shoulder pain to a general practitioner or a physiotherapist were included. Diagnostic codes were used for selection, and the cases were manually controlled. The cost for sick leave was calculated according to the human capital approach. Sensitivity analysis was used to explore uncertainty in various factors used in the model.

**Results:**

204 (103 women) patients, mean age 48 (SD 11) years, were registered. Half of the cases were closed within six weeks, whereas 32 patients (16%) remained in the system for more than six months. A fifth of the patients were responsible for 91% of the total costs, and for 44% of the healthcare costs. The mean healthcare cost per patient was €326 (SD 389) during six months. Physiotherapy treatments accounted for 60%. The costs for sick leave contributed to 84% of the total costs. The mean annual total cost was €4139 per patient. Estimated costs for secondary care increased the total costs by one third.

**Conclusions:**

The model applied in this study provides valuable information that can be used in cost evaluations. Costs for secondary care and particularly for sick leave have a major influence on total costs and interventions that can reduce long periods of sick leave are warranted.

## Background

Shoulder pain is a common cause of lost work days and disability. A majority of the patients are treated in primary health care [1-3]. In Sweden, health and medical care are organised in three levels: regional medical care, county medical care, and primary care which is organised by the county councils. Primary care is intended to meet the needs of most patients for medical treatment, care, preventive measures and rehabilitation. When more specialised care is necessary, patients are referred to the county hospitals. The regional hospitals treat rare and complicated cases. There were very few private care providers in the county at the time of this study. Resources are scarce, and the Swedish Health and Medical Services Act states that priority should be given to those who are in the greatest need of health and medical care. Quality of care can be defined as a combination of structure, process, and outcome [4]. Cost-of-illness studies can provide information about healthcare resources and costs allocated to different groups of patients.

Net costs to healthcare authorities for health and medical care in Sweden in 2005 were 16% for primary care and 52% for specialised physical care [5], most of which is financed from tax revenues. There is a government-imposed patient's cost ceiling for health care, meaning that no patient needs to pay more than €100 during a 12-month period, and no patient needs to pay more than €200 for prescription drugs covered by the benefits.

About 6,500 shoulders were operatively treated in Sweden in 2004 [6], and since 1998 the number of shoulder surgeries has increased by about 10% annually. A recent study reported a four-fold increase in the number of acromioplasties for rotator cuff disorders in New York State from 1996 to 2006 [7]. Multifactorial reasons were suggested for this increase, with patient-based, surgeon-based, and systems-based factors all playing a role. The differential diagnoses for shoulder pain are based on the history, acute or chronic nature of the pain, physical examination, and, if needed, completed with imaging. Tests for diagnostic accuracy [8] as well as surgical indications, are being discussed [1,9,10]. Although evidence from case series supports the effectiveness of surgical interventions for shoulder pain when used appropriately [1], the increase in shoulder surgery cannot be explained by the practice of evidence-based medicine. Three randomised clinical trials [11-13] comparing supervised exercises for subacromial pain with surgery, have concluded that supervised exercises are equally effective as surgery - and less expensive. One additional study found that only 10% of the patients awaiting surgery were finally operated on after being treated with physiotherapist-supervised exercises in a hospital setting [14]. This indicates a need for economic evaluations of current treatment strategies in primary health care.

The initial steps taken to diagnose and treat the patient in primary care may be essential for effective treatment, and may contribute to fewer patients being referred to surgery as well as lower costs to society. Kuijpers et al [15] reported costs of shoulder pain in primary care patients who presented with shoulder pain to their general practitioner (GP) in the Netherlands in 2006. Patients were followed for six months and their shoulder pain related costs were calculated by using patients' cost diaries. The patients reported all expenses relevant to their shoulder complaints; direct costs, such as visits to healthcare centres, and indirect costs, such as sick leave, and paid and unpaid help. In their study, 70% had persistent symptoms after six weeks and 46% after six months. They found that 12% of the patients with shoulder pain were responsible for 74% of the total costs, mostly a result of sick leave from paid work. Our study was performed to investigate the situation in Swedish primary health care, using an alternative design.

In Sweden, electronic patient records (EPR) based on diagnostic codes are used mainly in the clinical care of patients and rarely to evaluate healthcare programmes or cost-effectiveness aspects. Completeness and accuracy of diagnostic codes have been found acceptable [16,17], in spite of a coding system poorly adapted to primary health care. Attempts have been made, using EPR, to monitor the burden of illness for patients with low back pain [18], diabetes [19], and groups of patients according to their health status [20]. Linking costs and consequences based on already collected patient data may be useful to monitor the cost of illness in selected groups of patients.

The aim of this study was to assess the costs associated with healthcare use and loss of productivity caused by shoulder pain in Sweden, by auditing data from the EPR.

Questions asked in the study:

- What are the shoulder pain related treatment costs in primary care consulters in Sweden (direct costs)?

- What are the costs of shoulder pain in defined sub-groups of the selected population (highest costs)?

- What are the costs for sick leave (indirect costs)?

- What are the total costs?

## Methods

### Setting

The study was performed in 2009 in two municipalities, comprising 24 000 inhabitants, in a prosperous region on the Swedish west coast. The labour market in this region is based on trade and tourism, as well as many small and medium-sized enterprises. Three primary healthcare centres with three adjacent physiotherapy units were responsible for almost all primary health care in the area. There were few private alternatives to physiotherapy and no private physicians, making it possible to capture almost all patients who presented with shoulder pain in primary health care. In western Sweden, patients do not need a referral for physiotherapy. Sick leave for more than eight days must be prescribed by a doctor, although some employers require this from day one. The inclusion of patients was based on EPR in primary health care. We included all patients that presented with shoulder pain to any of these six units during the measurement period of six months, regardless of trauma or other diseases. Patients being permanent residents in either of the two municipalities and between 20 and 64 years of age were included, if any of the diagnostic codes given at the visit qualified them.

### Costing

A prospective cost-of-illness study was performed to explore the most important cost components of treating shoulder pain in primary health care. Healthcare costs and total costs, including cost for sick leave, were assessed. Costing involves identifying, measuring and valuing all resource changes that occur as certain healthcare interventions are carried out. In a bottom-up approach, individual elements are specified in detail. The three steps of the costing procedure in this study were:

1. Identification of relevant cost-items

2. Quantification of the use of the identified cost-items

3. Valuing the identified items

### Electronic patient records (EPR)

With very few exceptions, all units in primary health care in Sweden are computerised, and several EPR systems are in use. The data collected from the EPR were organized in a data matrix containing patients' personal identity number, age, sex, dates of encounter and diagnostic codes for every visit, number of admissions and referrals to specialist care, x-rays, number of drug prescriptions and sick leave periods prescribed by a GP. Our first step was to retrieve all visits to general practitioner or physiotherapist (PT) caused by shoulder pain during the measurement period. All data were anonymised before analysis.

At all participating units, notice boards were used to inform the patients. Receptionists were also asked to leave information sheets to patients who sought treatment for shoulder pain. All inhabitants in the area had been told that information from their EPR could be accessed and processed without consent for planning and quality assurance. The procedures of this study were approved by the Regional Ethical Review Board of Gothenburg.

### Management of shoulder pain

The Swedish guidelines for the management of shoulder pain [6] are similar to those for GPs in other countries [15,21-23]. Conservative (non-operative) care is recommended, including information on the prognosis of shoulder pain and advice regarding physical activities. In addition, the guidelines recommend a step-by-step treatment progression, consisting of physiotherapy treatment, pain relief and glucocorticoid injections (administered with or without local anaesthetic). If conservative treatment fails to reduce the symptoms, the patient is referred to an orthopaedic surgeon. In the present study physiotherapy treatments were adapted to each patient's condition and supervised exercises were emphasised.

The local hospital has a radiology department providing ultrasound evaluation of suspected tendon ruptures. MRI is regarded as a tool for orthopaedic surgeons and is seldom used in primary care in this part of Sweden.

The diagnostic coding system International Classification of Disease, version 10 (ICD-10), was used. Initially, a pilot study was performed at all six participating units to find out which diagnostic codes that were used for patients who consulted for shoulder pain. Fractures and dislocations of the shoulder were included. All visits with known and potential codes for shoulder pain were retrieved from the EPR system. Each individual with a potential code was scrutinized by comparing data within the EPR to verify the cause of visit. In the last step, 29 codes were classified in four categories, presented in Table 1: subacromial pain (including nonspecific shoulder pain), stiffness (adhesive capsulitis, arthritis), dislocations, and fractures.

**Table 1:** Diagnostic codes.

Subgroups	Diagnostic codes	Patients N (%)	Age (years) Mean (SD) Median	Sex: Male N (%)	Had Surgery N (%)
Subacromial pain	M751-9, M759P, M709, M779, M791, M799, M255, M255B, M629, M795, M796B	181(89)	48 (11) 51	89(49)	9 (48)

Stiffness	M750, M190B, M192B	10 (5)	52 (10) 54	7 (70)	2 (10)

Fractures	S420, S4200, S429	7 (3)	48 (14) 52	3 (42)	6 (32)

Dislocations	S430, S431, S435, S460	6 (3)	51 (13) 55	2 (33)	2 (10)

According to International Classification of Diseases, version 10 (ICD-10), used for shoulder pain and merged into four categories. Code names are presented below. All patients, N = 204. Patients who had surgery or other orthopaedic intervention, N = 19

M75. Shoulder lesions; M750 Adhesiv capsulitis

M70-M79 (B) Other soft tissue disorders (shoulder); M629 Disorder of muscle

M255 (B) Pain in joint (shoulder); M19.(B) Arthrosis (shoulder)

### Procedure

The cost-of-illness calculation was based on all registered actions related to shoulder pain during the measured period. Patients referred to orthopaedic surgeon for evaluation were followed up to monitor whether they were selected for surgery or not.

Total treatment time and sick leave at inclusion were retrieved from the EPR. The period between first and last dates of visit to a GP or a PT with the qualifying code was defined as the total treatment time. At least one visit per month had to be registered, except during the holiday period.

Half of the patients started and ended treatment within six months. Some started before and some ended after the measured period. We believe that this would be the case at any chosen period during the year. Costs for all patients passing through during six months can be multiplied by 2 to estimate the annual cost for this group of patients. This estimate can then be used to compare with annual costs in other regions. This method is also suitable to investigate the relative size of the different treatment components.

Calculation of treatment costs per patient requires complete registration of all activities during the whole treatment period. Patients must be monitored from their first encounter for shoulder pain, although onset may be difficult to define. They should preferably be monitored for a long time period, ideally for the rest of their lives.

### Valuing healthcare costs

Costs used in the economic evaluation are presented in Table 2. Healthcare costs per visit to GP were in our study set at €107. This figure was based on reports from the National Board of Health and Welfare, in which the cost was calculated to €92 in 2004, costs for medication and medical services *excluded*. To this we added an annual increase in costs of 3%. We compared this with the local inter-county price list in Sweden for 2009 [24], where a visit to GP, *including *x-ray, medication and laboratory services, was charged with €124. From these figures we found our estimate per visit to be appropriate. We used the cost for physiotherapy treatment, €50, from the same inter-county price list, since no other figures were available. Charges to primary care for x-ray and ultrasound evaluations were retrieved from the hospital's radiology department.

**Table 2:** Costs used in the economic evaluation

Costs	(Euros*)
*Direct healthcare costs (per visit)*	

General practitioner (25 min)	107

Physiotherapist (60 min)	50

x-ray, shoulder	65

Ultrasonography, shoulder	124

Medicine	Prices July 2009

Orthopaedic specialist	335

MRI	308

Shoulder surgery, uncomplicated, ambulatory care	2420

*Indirect costs*	

Sick leave from paid work (human cost method) per day	205

*1 Euro = 10.62 SEK. Average values in 2009, http://www.riksbank.se (Swedish National Bank)

Medication prescribed during the registered visits was retrieved from the EPR. Medication purchased without prescription was not registered. Costs for analgesics and nonsteroidal anti-inflammatory drugs were calculated as if every prescription was filled once and as if the patient had free medication, meaning that the costs were paid by the primary care unit. Costs were retrieved from the hospital pharmacy.

Patients who were referred to an orthopaedic specialist and to surgery generated additional costs. From the local inter-county price list [24] and the hospital administration we retrieved the costs for visits in outpatient care and a mean cost for ambulatory surgery in 2009, based on actual costs per patient. We estimated that ten MRI investigations would be performed in the patients evaluated in the present study. These figures illustrate the higher costs for secondary care (Table 2).

### Valuing productivity costs

The costs for sick leave were for the baseline value calculated according to the human capital approach [25,26]. This method places monetary weights on healthy time using market wage rates. It is an estimation of changes in productivity, based on the opportunity cost of the production that people would have contributed to, had they been at work. We assumed that the production costs were reflected by the salary. In this study, we only had data on sick leave periods (graded from 25 to 100% of full working time) prescribed by GPs. Partial sick leave was converted to 100% sick leave for each patient. The cost per day was calculated from the mean income in the region in 2008, provided by the Swedish Bureau of Statistics. The costs for productivity loss due to sick leave were calculated after this model presented by the Swedish Ministry of Industry in 2001 [27]:

Costs for productivity loss = Mean income + social fares + indirect taxes.

We assumed that social fares were 40% of the main cost and indirect taxes were 28%.

This equation shows what the worker must produce to cover his own income, payroll taxes and fees by law and agreement.

### Human capital versus friction cost method

An alternative approach to the human capital method is the friction cost method [25,28]. In that case we assume that when a person has a period of sick leave, there is a pool of unemployed people that can replace the sick person. Hence, there will only be a productivity loss in a "friction" period until the new employee is recruited and trained to do the job. It is frequently argued that evaluations using the human capital approach overestimate the true costs to society [25]. Koopmanschap et al [29] found that cost of absence from work in 1988 when using the friction cost method was 38.7% of what they found by using the human capital approach. The cost for disability was 0.3% and for mortality 1.9% if the friction cost method was used. As part of the sensitivity analysis we displayed the effect of using the friction cost instead of the human capital method.

### Data analysis

Costs were calculated for a six-month period. The arithmetic mean, standard deviations (SD), and median value were used to provide information about the total cost of treatment for all patients, and to illustrate the skewness in the distribution of costs and resource use. The total costs during six months, were multiplied by 2 in order to get the total annual costs for patients with shoulder pain in primary health care.

One-way and two-way sensitivity analyses were performed to explore the uncertainty [30], to demonstrate the impact of one parameter varying in the model, and to examine the relationship of two or more different parameters changing simultaneously.

We used a multivariable linear regression analysis to explore how gender, age and municipality, as independent variables, predicted costs.

## Results

### Patients

During six months 204 patients were registered; 103 women and 101 men. Mean age was 48 (SD 11) years. Eighty-nine per cent presented with subacromial or nonspecific shoulder pain (Table 1). Nineteen patients (9%) came for postoperative rehabilitation. Twenty-nine patients (14%) were referred to an orthopaedic surgeon, and four of these (2%) went on to have surgery within a year. Fifty per cent of the cases were closed within six weeks, whereas 32 patients (16%) remained in the system for more than six months. Seven of these patients had been operated on. Baseline characteristics of the group are presented in Table 3.

**Table 3:** Baseline characteristics of patients with shoulder pain.

Characteristics	n = 204	n = 45*	n = 19**
Age (years); mean (SD)	48 (11)	48 (11)	48 (13)

Sex: male; n (%)	101 (49)	20 (44)	10 (51)

Treatment duration of current shoulder complaints***			

0-6 weeks	101 (50)	15 (33)	4 (21)

7-12 weeks	28 (14)	4 (9)	2 (10)

12-26 weeks	42 (21)	12 (29)	6 (32)

> 6 months	33 (16)	14 (29)	7 (37)

Duration of sick leave in the 8 weeks preceding inclusion****			

0 weeks	193 (95)	37 (82)	17 (89)

0-1 weeks	4 (2)	3 (7)	1 (5)

1-8 weeks	7 (3)	5 (11)	1 (5)

Numbers (percentages) are presented unless stated otherwise

* patients generating costs of > €1000 in 6 months

** had shoulder surgery

*** total treatment time for all patients. Costs calculated for 6 months

**** sick leave due to shoulder pain

### Use of healthcare resources and sick leave

Consumption of healthcare resources and sick leave from work during six months are presented in Table 4. Forty patients (20%) had a period of sick leave prescribed by GP, mean 9.0 days (SD 29.2). Three patients (1.5%) were on sick leave due to their shoulder pain for more than six months; two of them with concomitant back pain and one with concomitant diabetes. Partial sick leave amounted to 11% (202 days) during the measured period.

**Table 4:** Costs (€) and consumption of healthcare resources and sick-leave during 6 months.

Direct costs	Mean number of visits	Total number	Cost per patient	Total costs
General Practitioner	0.89 (0.97)	181	95 (105)	19429

Physiotherapy	3.91 (7.40)	798	195 (369)	39825

X-ray*	0.28 (0.45)	57	18 (29)	3719

Ultrasound*	0.11 (0.31)	23	14 (39)	2857

Medicine*	0.28	58	4 (6)	718

**Total healthcare costs**			**326 (389)**	**66548**

**Indirect costs**				

Sick-leave**	9.04 (29.17)	1844	1743 (5626)	**355610**

**Total costs**			**2069 (5730)**	**422158**

N = 204. Means (SD) or total numbers are presented

* Number of patients given prescriptions for medicine, x-rays or ultrasound

** Days

Fifty-five patients (27%) consulted both a GP and a PT within 4 weeks. Nineteen patients (9%) had more than 10 physiotherapy treatments, 68 patients (33%) had none. The whole group had a mean of 6.7 (SD 7.0) physiotherapy treatments and 24.0 (SD 50.2) days of sick leave.

The consumption of medication, x-ray and ultrasound evaluations was low.

### Costs

Costs for healthcare use and sick leave are presented in Table 4. The mean healthcare cost per patient was €326 (SD 389). Physiotherapy treatments accounted for 60%. This cost was twice as high as for visits to GP. The group of 73 patients that used the direct access to PT incurred a higher mean total cost for physiotherapy but lower healthcare and total costs.

The healthcare costs for the group with persistent symptoms were one fourth of all healthcare costs during six months. Median healthcare costs were €200 (Inter Quartile Range 113-397) for the whole group, whereas the median total costs were €249 (IQR 119-661). Eighty-four per cent of the total costs were due to sick leave prescribed by GP, for the whole group and for those who had surgery.

**Figure 1:**
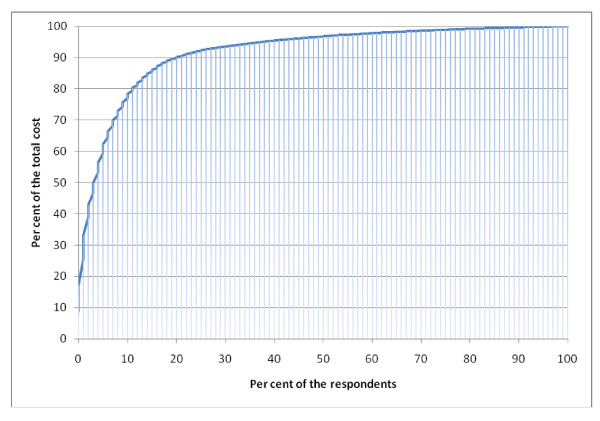
**Distribution of total costs**.

**Figure 2:**
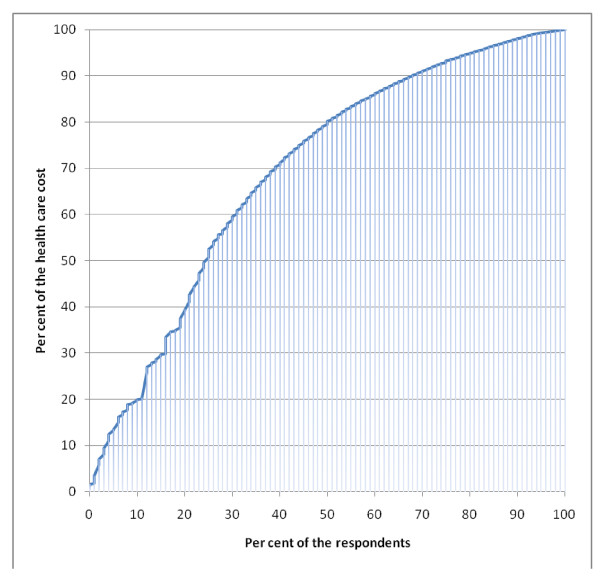
**Distribution of total healthcare costs**.

Total costs for the 45 patients (22%) with costs > €1000 during six months are presented in Table 5. Sick leave in this group amounted to 91% of the total costs, and for 44% of the healthcare costs (Figures 1 and 2). Seven patients in this group had no registered sick leave. Eighteen patients had symptoms for more than 6 months; five of them had no registered sick leave. The three patients with sick leave > 6 months contributed to 25% of the total costs.

**Table 5:** Costs (€) and consumption of healthcare resources and sick-leave during 6 months for the group that cost > €1000.

Direct costs	Mean number of visits	Total	Cost per patient	Total costs
GP	1.71 (1.42)	77	184 (153)	8266

PT (cost per visit)	8.20 (13.62)	369	409 (680)	18415

X-ray*	0.33	15	22 (31)	979

Ultrasound*	0.27	12	33 (56)	1490

Medicine*	0.47	21	6 (7)	262

**Total healthcare costs**			**654 (671)**	**29412**

**Indirect healthcare costs**	Days			

Sick-leave**	40.82 (50.98)	1837	7875 (9833)	**354356**

**Total cost**			**8528 (9829)**	**383768**

N = 45. Means (SD) or total numbers are presented

* Number of patients given prescriptions for medicine, x-rays or ultrasound

** Days

The mean annual total cost for patients with shoulder pain in primary health care was €4139 per patient. Additional healthcare costs were generated by 29 patients (14%), MRI investigations, and from four cases of surgery in ambulatory care. The costs for secondary care for this group were estimated at €22475, corresponding to one third of the total costs for primary care.

### Uncertainty

To show the uncertainty of the results we have reported the 95% confidence interval (CI) for the base case scenario for total costs and for healthcare costs in Table 6. The CI is €1 283- 2856 and €273-380, respectively. These intervals reflect the uncertainty caused by the fact that different patients use services such as x-ray and PT consultations with different *frequencies*. Additional uncertainty is related to the *cost per unit *of health services and the cost of sick leave per day. To show the importance of this uncertainty we performed a sensitivity analysis. For x-ray cost per examination we chose as an example +30% as a maximum average value and -30% as a minimum value. For each tested parameter value we computed the new expected costs and 95% CI based on the sample variation related to the (unchanged) frequencies of health service use and the new cost level per unit. The sensitivity analysis showed that the total cost was most sensitive to the choice of method for estimating the sick leave cost. Compared to the base case scenario where we used the human capital method, the friction cost method gave a reduction of the total cost per patient of 51.6% to €1001. Because of the dominance of the sick leave cost, the reasonable change of healthcare cost has just a minor influence on the total cost. A 30% change in physiotherapy cost or a 50% change in physician cost contributes just to a 2.8 and 2.3% change in the total cost, when changing these parameters one by one (one-way sensitivity analysis). When changing all the parameters of health service cost in the same direction (multi-way sensitivity analysis) as shown in Table 6, the total cost only changes by 5.7%.

**Table 6:** Uncertainty.

Changed parameter or method	Percentage change in parameter	Percentage change in total cost	Percentage change in HC cost	Total cost (95% confidence interval)	Healthcare cost (95% confidence interval)
Base case scenario	0.0	0.0	0.0	2069 (1283-2856)	326 (273-380)

**Parameters, one-way sensit. analysis**					

PT cost per consultation	+30	2.8	18.0	2128 (1339-2917)	385 (317-453)
	-30	-2.8	-18.0	2011 (1227-2795)	268 (228-307)

GP cost per consultation	+50	2.3	14.6	2117 (1328-2906)	374 (318-430)
	-50	-2.3	-14.6	2022 (1238-2805)	279 (227-330)

Sick leave cost per day	+30	25.3	0.0	2592 (1575-3610)	326 (273-380)
	-30	-25.3	0.0	1546 (991-2102)	326 (273-380)

X-ray cost per consultation	+30	0.3	1.7	2075 (1288-2861)	332 (278-385)
	-30	-0.3	-1.7	2064 (1278-2850)	321 (268-374)

Ultrasound cost per consultation	+30	0.2	1.3	2074 (1287-2860)	330 (277-384)
	-30	-0.2	-1.3	2065 (1279-2851)	322 (269-375)

Medicine, unit used	+100	0.2	1.1	2073 (1286-2859)	330 (276-383)
	-50	-0.1	-0.5	2068 (1281-2854)	324 (271-378)

**Parameters, multi-way sensit. analysis**					

PT, GP per consultation	+30. +50	5.1	32.6	2176 (1384-2967)	432 (364-502)
	-30. -50	-5.1	-32.6	1963 (1182-2744)	220 (183-257)

PT, GP, x-ray, ultras. per consultation	As for one- way sensit. a.	5.7	35.8	2186 (1394-2979)	443 (373-513)
		-5.7	-35.8	1952 (1172-2733)	209 (173-246)

**Method, one-way sensitivity analysis**					

Sick leave cost based on friction method	-61.3	-51.6	0.0	1001 (685-1316)	326 (273-380)

Calculation of percentage change and new levels of total costs and health service costs by changing the unit costs of the different cost components

The sensitivity analysis showed that the physiotherapy unit cost makes the biggest contribution to uncertainty in the health service cost. A 50% change in the physician unit cost changes the health service cost by 14.6%, and a 30% change of physiotherapy costs gives a change of 18.0%. Relevant changes in the costs of x-ray, ultrasound and medicine only have a minor influence.

Gender, age and place of treatment did not influence total costs or health service costs. A sensitivity analysis using the logarithm of total cost and health service cost did not change this conclusion (Table 7).

**Table 7:** Multivariable linear regression analysis

Independent variables	Log (total cost)	Log (health service cost)
Place of treatment	-0,043	-0,119
	(0,118)	(0,224)

Gender	0,160	0,202
	(0,117)	(0,222)

Age	0,003	-0,002
	(0,005)	(0,010)

Constant	5,207	6,007
	(0,275)	(0,523)

Observations	203	203

The numbers in parentheses below the estimates are the standard errors

## Discussion

The main finding in the present study is that the mean healthcare costs amounted to less than 20% of mean total costs for patients with shoulder pain. Contrary to this, median healthcare costs contributed to 80% of median total costs, reflecting a minority of patients incurring high costs from long lasting sickness absence. Our findings are in keeping with previously published results on patients with shoulder and back pain [15,18,31].

### Treatment strategies

The majority of patients were managed in primary care. Fifty per cent were treated within six weeks, and only two per cent were selected for surgery. This is in line with the intentions in guidelines and literature. Surgery should be considered if it represents an evidence-based approach when conservative measures fail. A treatment strategy for patients with subacromial pain is currently evaluated [32]. The observed increase in shoulder surgery does not correspond with a similar increase in prevalence of shoulder pain [33]. Vitale et al [7] discussed the increasing utilization of surgical procedures overall in recent years, and Hofmann [34] argued that there is a technological imperative in health care.

The inter quartile range of total costs varied from 119 to 661, illustrating the impact of long periods of sick leave. A fifth (22%) of the population generated costs of more than €1000 and accounted for 91% of the total costs. In the Dutch study [15], 12% of the patients cost more than €1000 and contributed to 74% of the total costs. The three patients with sick leave > 6 months contributed to 25% of the total costs. Efforts have been made to reduce long periods of sick leave, often combined with programmes for pain management [35-37]. Multidisciplinary rehabilitation programmes for patients with chronic low back, neck or shoulder pain are reported to be superior to treatment as usual for return to work [38,39]. However, a Cochrane review [40] on the subject did not find evidence to recommend multidisciplinary rehabilitation for patients with neck and shoulder pain. In the present study, physiotherapy treatments accounted for 60% of the healthcare costs and two thirds of the patients consulted a PT 3-4 times on average.

Whether an intervention programme is cost-effective or not depends on the relevance of the clinical outcomes and the costs needed to achieve this [41-43].

In the present study, 89% were diagnosed with subacromial or nonspecific shoulder pain. Feleus et al [44] found that 41% of the patients with non-traumatic neck, arm, or shoulder pain were given an unspecific diagnostic code at the first consultation in primary health care, and no differences were found in severity, complaints or functional limitations compared to patients with a specific diagnostic code. A specific diagnosis was given in 59% of the cases, mostly subacromial impingement syndrome. Distinction between diagnostic groups is important if these groups have different prognoses or require different management. Patients with specific diagnoses were more frequently referred for specialist treatment, while patients with non-specific diagnoses were more frequently referred for physiotherapy in the Dutch study [44]. Nonspecific shoulder pain - the presence of pain without specific physical signs and pathology - is common, and Miranda et al [45] found that subjective complaints without clinical findings may indicate adverse psychological and psychosocial factors rather than an underlying pathologic condition. Several studies have reported that long-term sickness absence was associated with work conditions rather than with individual characteristics [46].

Future studies should include cost-effectiveness evaluation of various physiotherapy regimens or comparisons of physiotherapy with other treatments for shoulder pain. Functional limitations and duration of sick leave should be included as outcome measures. Such studies will be extensive and time-consuming, but study protocols have been presented [47,48].

### Strengths and limitations of the study

A limitation of the present study is that we do not know whether the patients were relieved from their symptoms when the treatment period was ended, or if they disappeared out of the system for other reasons. The costs were limited to the primary diagnoses for the visit, and ignored costs associated with comorbidity. This is often the case in cost-of-illness studies and a simplification of real life, as has been pointed out by Koopmanschap [49]. When we looked closer into the three cases with sick leave more than six months, we found that they all had additional diagnoses. We could not gather such information for the rest of the group with the method applied.

We had information about sick leave periods prescribed by GP, but we do not know if patients were actually absent from work all that time. We had no information about short-term sick leave, nor whether patients had sick leave prescribed by orthopaedic surgeon post-operatively. To fully estimate the cost for productivity loss additional data would have been required, for instance self-reported data from cost diaries or log-books [15,42], or questionnaires [32]. However, a recent study suggests that self-reported data are less valid than register-based data to measure the number of days on sick leave [50].

The cost for medication is probably underestimated in this study. We had no information on the consumption of drugs, nor of the medication paid out of pocket. However, medication had a minor contribution to the total cost, and we do not expect that costs for medication would have an important impact on the results.

Generalization to other settings might be difficult, and will depend on how diagnostic codes are used, how reliable the registration is, and how costs are determined. The reliability of the cost estimates and varying research methodology have been under debate [51]. Charges for hospital services, like radiographic imaging, do not always reflect the actual unit cost of a production, but is merely a vehicle for transferring money between healthcare service units. However, these costs are easily available and most often the only costs available and therefore used in the present study. The measurement of productivity loss due to illness is highly dependent of the choice of approach, and this calls for standardisation on a national level. In the Netherlands a "Standardisation of costs; a manual for costing in economic evaluations" [52] was issued to eliminate some of the price differences between studies and to give guidelines for a uniform costing methodology.

The strength of the present study is that we were able to capture almost all patients consulting with all types of shoulder pain during a six-month period. There were few alternatives to medical care and data were manually controlled. We can double the total cost to illustrate the annual cost to society and to the health care system for shoulder pain in the chosen area. Our study provides direct and meaningful information about the size of the problem and can be an essential component in further cost-effectiveness analyses of different treatment strategies in primary health care.

## Conclusions

Costs for sick leave for shoulder pain contributed to more than 80% of the total costs for society for this patient category. These results are in line with other studies on neck, shoulder and back pain. Health care interventions should focus on getting people back into the workforce, with special attention towards the small group that generates the highest costs. The model applied in the current study may be applied in future studies to analyse changes over time in terms of illness patterns in medical and health economic perspectives. A societal perspective is needed for the inclusion of all consequences of the interventions.

## Competing interests

The authors declare that they have no competing interests.

## Authors' contributions

LV, PJ and RE designed the study. LV collected the data. All authors participated in the study, and drafted the manuscript. PJ performed the statistical analysis. All authors read, critically revised, and approved the final manuscript.

## Pre-publication history

The pre-publication history for this paper can be accessed here:

http://www.biomedcentral.com/1471-2474/13/17/prepub
